# Pro-protein convertases control the maturation and processing of the iron-regulatory protein, RGMc/hemojuvelin

**DOI:** 10.1186/1471-2091-9-9

**Published:** 2008-04-02

**Authors:** David Kuninger, Robin Kuns-Hashimoto, Mahta Nili, Peter Rotwein

**Affiliations:** 1 Department of Biochemistry and Molecular Biology, Oregon Health & Science University, Portland, Oregon, 97239-3098, USA

## Abstract

**Background:**

Repulsive guidance molecule c (RGMc or hemojuvelin), a glycosylphosphatidylinositol-linked glycoprotein expressed in liver and striated muscle, plays a central role in systemic iron balance. Inactivating mutations in the RGMc gene cause juvenile hemochromatosis (JH), a rapidly progressing iron storage disorder with severe systemic manifestations. RGMc undergoes complex biosynthetic steps leading to membrane-bound and soluble forms of the protein, including both 50 and 40 kDa single-chain species.

**Results:**

We now show that pro-protein convertases (PC) are responsible for conversion of 50 kDa RGMc to a 40 kDa protein with a truncated COOH-terminus. Unlike related molecules RGMa and RGMb, RGMc encodes a conserved PC recognition and cleavage site, and JH-associated RGMc frame-shift mutants undergo COOH-terminal cleavage only if this site is present. A cell-impermeable peptide PC inhibitor blocks the appearance of 40 kDa RGMc in extra-cellular fluid, as does an engineered mutation in the conserved PC recognition sequence, while the PC furin cleaves 50 kDa RGMc *in vitro *into a 40 kDa molecule with an intact NH_2_-terminus. Iron loading reduces release of RGMc from the cell membrane, and diminishes accumulation of the 40 kDa species in cell culture medium.

**Conclusion:**

Our results define a role for PCs in the maturation of RGMc that may have implications for the physiological actions of this critical iron-regulatory protein.

## Background

Iron is an essential element required for many cellular processes, including energy metabolism, oxygen transport, and respiration [1]. Iron homeostasis is tightly regulated, and there are major health consequences linked to both its deficiency and excess [1] Normal iron homeostasis is disrupted in hemochromatosis, a heterogenous hereditary disorder of iron overload. Juvenile hemochromatosis (JH) is a rapidly progressive form of this disease with severe systemic consequences if untreated [3]. Many patients with JH have mutations in the HJV gene, which encodes hemojuvelin [4-7], and mice lacking *hjv *develop an iron-overload phenotype [8,9]. Hemojuvelin is identical to RGMc, and with RGMa and RGMb, comprise the repulsive guidance molecule (RGM) family [4,10,11]. RGMa and b are produced primarily in the central nervous system [11,12], and play roles in neuronal survival and patterning [11,12], while RGMc is synthesized in liver and striated muscle [10,11,13,14]. All three RGM genes encode glycosylphosphatidylinositol-anchored and soluble glycoproteins. For RGMc, these consist of single-chain and heterodimeric membrane-linked molecules, and soluble 50 and 40 kDa single-chain proteins that arise from an incompletely defined biosynthetic and processing pathway [14-17].

The mechanisms by which RGMc participates in systemic iron balance are unknown. The liver-derived hormone, hepcidin, is an essential regulator of iron homeostasis that acts by controlling intestinal iron absorption and recovery from macrophages [1]. Hepcidin binds to the membrane iron transporter, ferroportin, leading to its degradation [2]. In hemochromatosis, hepcidin levels are low, and dietary iron uptake is excessive [3]. Recent studies have suggested that membrane-associated RGMc increases hepcidin gene expression in the liver by collaboration with signaling pathways activated by bone morphogenic proteins (BMP) 2 and 4 [18,19], and thus acts to prevent iron import. By contrast, soluble RGMc may inhibit hepcidin synthesis [15,20]. RGMc also may promote iron uptake into cells [16], but biochemical mechanisms have not been defined.

Here we demonstrate a role for pro-protein convertases (PC) in the biogenesis of RGMc, and in their regulation by iron. Through biochemical and cell-based approaches we show that PCs cleave full-length 50 kDa RGMc at an evolutionarily conserved recognition site into a 40 kDa soluble species truncated at its COOH-terminus. Both 50 and 40 kDa RGMc are found in the blood of humans and mice, and in extra-cellular fluid of cultured cells. The relative ratio and overall abundance of both RGMc species appears to be altered by cellular iron levels, with iron loading leading to a decline in soluble RGMc, but an increase in the 50 kDa isoform and in the amount of single chain RGMc retained on the cell membrane. Thus our results define potential interactions between PCs and iron to control the expression of a critical iron-regulatory protein.

## Methods

### Cell culture

All cells were incubated at 37°C in humidified air and 5% CO_2_. The following established cell lines were used. Murine C3H10T1/2 cells (ATCC #CCL-226, Manassas, VA, USA) and C2 myoblasts [14] were grown on gelatin-coated dishes in DMEM (Mediatech-Cellgro, Herndon, VA, USA) plus 10% heat-inactivated fetal calf serum (FCS, Hyclone, Logan, UT, USA). C3H10T1/2 cells were infected at ~50% of confluent density with a recombinant adenovirus encoding MyoD, as described [14], and muscle differentiation-promoting medium (DMEM and 2% horse serum (Hyclone)) was added 24 h later. C2 myoblasts were incubated at confluent cell density in muscle differentiation-promoting medium, as described [14]. Cos-7 (ATCC #CRL-1651) and Hep3B cells (ATCC #HB-8064) were grown in DMEM and 10% FCS. Ferric ammonium choride or the iron chelator deferoxamine (Sigma, St. Louis, MO, USA) was added to medium for 24 h.

### Recombinant adenoviruses

Ad-MyoD, Ad-tTA (tetracycline transactivator protein), Ad-HA-RGMc, and Ad-HA-RGMcΔGPI have been described [14,21]. At 18 h after viral infection, new medium was added (DMEM and 2% horse serum) containing the cell-impermeable pro-protein convertase inhibitor, decanoyl-Arg-Val-Lys-Arg-chloromethyl-ketone [10 μM] (RVKR, Alexis Biochemicals, San Diego, CA, USA) or DMSO. Cells and medium were harvested over the next 24 h. Hep3B cells were infected at ~50% confluent density with Ad-HA-RGMc or Ad-HA-RGMcΔGPI, and Ad-tTA, and treated similarly.

### Expression of RGMc mutants

We previously cloned a mouse RGMc cDNA from skeletal muscle cells [13]. The following codon changes were introduced into the cDNA by site-directed mutagenesis (Stratagene, San Diego, CA, USA): R318G, R321A, R324A. Mouse RGMc truncation mutants were made by PCR by replacing codons after R378, C354, S321 and Q305 with a 6× His epitope tag and stop codon. These alterations correspond respectively to human JH-associated frame-shift mutations R385X, C361fsX366, S328fsX337 and Q312X [4-7]. DNA sequencing was used to confirm all nucleotide changes, and the regions with mutations were subcloned into HA-RGMc in pcDNA3 [13]. Transient transfections were performed using 2 μg of DNA/35 mm dish, and RVKR or DMSO were added 18 h later. Cells and medium were harvested after an additional 24 h.

### Immunoblotting

Conditions for preparation of whole cell protein lysates and culture medium, SDS-PAGE, and immunoblotting have been described [14]. Primary antibodies included: mouse RGMc (1:750 dilution) [14], HA (Covance, Denver, PA, USA; 1:4000), α-tubulin (Sigma, 1:4000), His (Abcam, Cambridge, MA, USA; 1:1000), and pan-cadherin (Cell Signaling, Danvers, MA, USA; 1:1000). Secondary antibodies included Alexa 680-conjugated anti-mouse IgG (Molecular Probes, Eugene, OR, USA; 1:4000) and IRD 800-conjugated anti-rabbit IgG (Rockland, Gilbertsville, PA, USA; 1:4000).

### Purification of RGMc

An antibody affinity column was prepared by coupling 1.5 mg of antigen-purified rabbit anti-RGMc IgG to CNBr-activated Sephadex 4B (Amersham-Pharmacia, Piscataway, NJ, USA). Serum was obtained from two healthy humans (ages 25 and 35), and two male mice (age 10 and 12 weeks). Mouse or human serum (0.5 ml) or conditioned culture medium (1 ml) was diluted into 10 mM TrisHCl, pH 7.4, 0.05% Tween-20, and protease inhibitors (Roche, Indianapolis, IN, USA). Samples were pre-cleared with protein-A agarose (Sigma) for 4 h at 4°C, then incubated with affinity resin for 16 h at 4°C. Following washes (10 column volumes of 10 mM TrisHCl, pH 7.4, 0.05% Tween-20), proteins were eluted with 0.5 ml of 100 mM glycine, pH 2.5, and neutralized with 1 M TrisHCl, pH 8.0. A total of 50 μl was used for detection by SDS-PAGE and immunoblotting [14].

### Cell-surface biotin labeling

Monolayer cultures were incubated with EZ-link sulfo-NHS-biotin (1 mg/ml, Pierce, Rockford, IL) for 30 min at 4°C, followed by incubation in medium ± RVKR, 'pull-down' of protein extracts or culture medium with streptavidin-agarose, and SDS-PAGE and immunoblotting [14].

### Incubation of RGMc with cells or recombinant furin

Conditioned medium from cells expressing RGMc plus RVKR was dialyzed to remove inhibitor. Aliquots (200 μl) were added to Hep3B cells plus fresh RVKR or DMSO for 24 h at 37°C, followed by SDS-PAGE and immunoblotting. Dialyzed medium (25 μl) was incubated with 10 U recombinant human furin for 4 h at 30°C in 100 mM Hepes, pH 7.4, 1 mM CaCl_2_, and 0.5% Triton-X100, followed by SDS-PAGE and immunoblotting.

## Results

### A pro-protein convertase inhibitor prevents accumulation of soluble 40 kDa RGMc

RGMc is produced by hepatocytes and striated muscle [8,13,15]. We have detected RGMc on muscle cell membranes, and have found that proteins of ~50, 35, and 20 kDa are released into the extracellular fluid after incubation with bacterial PI-PLC [14], illustrating that RGMc is attached to the membrane by a GPI linkage. The two smaller protein bands comprise a disulfide-linked heterodimer, while the larger species is full-length single-chain RGMc [14]. Similar results have been observed for RGMc over-expressed in cell lines [14-16]. RGMc also accumulates in medium conditioned by muscle cells as 50 and 40 kDa single-chain proteins [14], suggesting either direct secretion or release from the plasma membrane.

**Figure 1:**
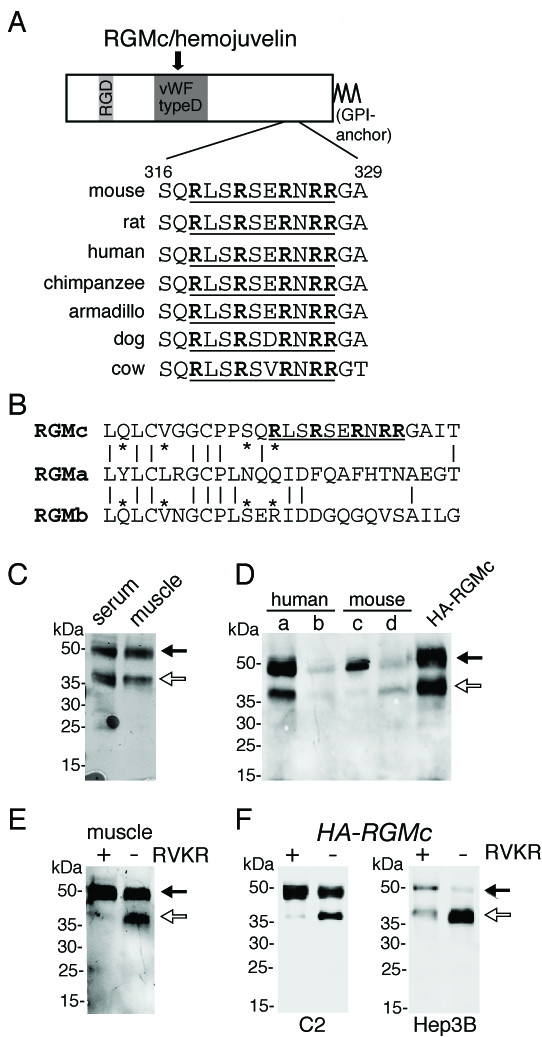
**A pro-protein convertase inhibitor prevents accumulation of soluble 40 kDa RGMc**. **A. **Map of mouse RGMc with RGD motif, von Willebrandt factor (vWF) type D domain, GPI anchor, and intra-molecular cleavage site (arrow) indicated. The conserved PC site (underlined, arginines **bolded**) is aligned below the map for 7 mammalian species. **B. **Absence of putative PC sites in RGMa or b. Alignment of human RGMa, b, and c; vertical lines or asterisks denote identical residues between paralogous proteins. **C. **Immunoblot of affinity-purified RGMc from mouse serum and muscle cell conditioned medium, with 50 and 40 kDa isoforms indicated. **D. **Immunoblot of affinity-purified RGMc from blood from two humans (a, b) and two mice (c, d), with 50 and 40 kDa isoforms indicated. Soluble HA-RGMc is included as a reference. **E. **Immunoblot of RGMc from muscle cell conditioned medium (50 μl) incubated without or with the small molecule PC inhibitor, RVKR. **F. **Immunoblot of RGMc from conditioned medium (25 μl) of C2 myoblasts or Hep3B cells infected with Ad-HA-RGMc and incubated ± RVKR. For **C **– **F**, black arrows indicate 50 kDa RGMc and white arrows 40 kDa.

Here we investigate mechanisms responsible for the appearance of 40 kDa RGMc in extra-cellular fluid. Full-length 50 kDa RGMc is a highly conserved protein, with ~76% homology among the 7 mammalian species shown in Fig. 1A, and many stretches of amino acid identity, including the arginine-rich segment found between residues 318 and 327 in mouse RGMc (Fig. 1A) that is not conserved in RGMa or RGMb (Fig. 1B). This motif resembles a recognition and cleavage site for pro-protein convertases (PC). These enzymes, PC1, PC2, furin, PC4, PC5, PACE4 and PC7 [22], cleave substrates at the COOH-terminal basic residue in the sequence (K/R)-(X)_*n *_-(K/R), where *n *= 0, 2, 4, or 6 amino acids and X is any residue except Cys or Pro [22]. To determine if full-length RGMc is a substrate for PCs, we first purified the soluble protein by antibody affinity chromatography from culture medium from differentiating muscle cells, and from mouse and human blood. Both 50 and 40 kDa RGMc species were detected from all three sources (Fig. 1C and 1D). Surprisingly, serum concentrations differed substantially between two healthy humans, and between two healthy male mice of the same age and on the same diet, where in one mostly the 50 kDa form of RGMc was detected (Fig. 1D, lane c vs. d). Further study will be required to assess the mechanisms responsible for this variation. When muscle cells, which produce RGMc [14], were incubated with the peptide PC inhibitor, RVKR, the 50 kDa species became the only RGMc protein in the medium (Fig. 1E), implicating PC activity in processing of endogenous RGMc. Similar results were seen in undifferentiated mouse C2 myoblasts and in human Hep3B cells expressing HA-RGMc (Fig. 1F), although it should be noted that the same dose of RVKR was less effective in Hep3B cells in preventing accumulation of 40 kDa RGMc, implying that there is more PC activity in this cell line.

### Mapping the location of PC cleavage by analysis of JH-associated RGMc frame-shift mutations

**Figure 2:**
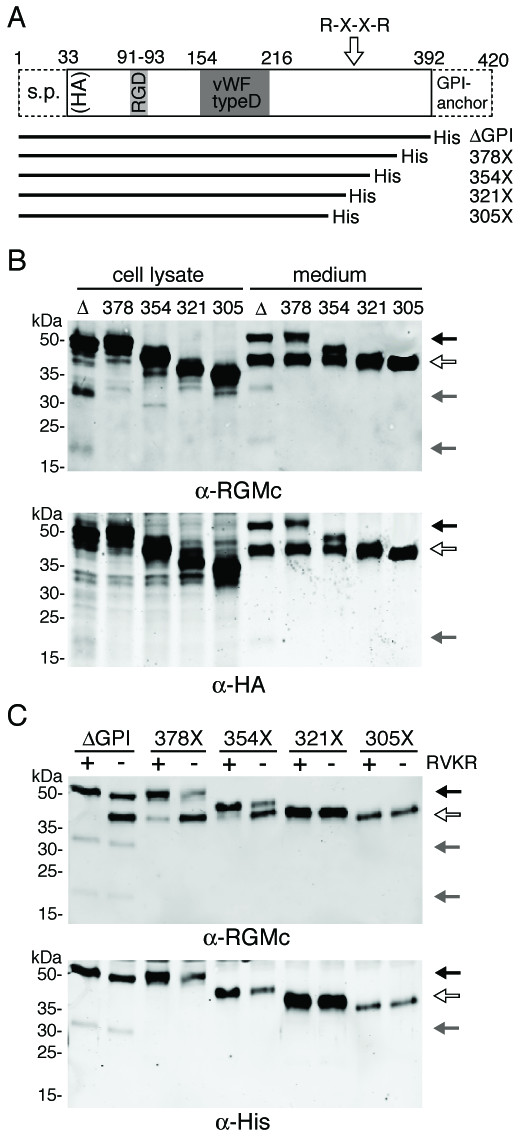
**Juvenile hemochromatosis-linked RGMc COOH-terminal truncation mutants are cleaved into smaller isoforms**. **A. **Map of mouse RGMc showing locations of signal peptide (s.p.), RGD sequence, von Willebrandt type D domain, GPI-anchor motif, and putative PC site (R-X-X-R). RGMcΔGPI-His and RGMc frame-shift truncation mutants are pictured below. Each contains a COOH-terminal His tag. **B. **Detection of RGMc truncation mutants by immunoblotting of protein lysates and conditioned medium from transiently transfected Cos-7 cells using RGMc (upper panel) or HA antibodies (lower). **C. **Detection of RGMc truncation mutants by immunoblotting of conditioned medium after incubation ± RVKR using RGMc (upper panel) or His antibodies (lower). For **B **and **C**, black arrows indicate 50 kDa single-chain RGMc and white arrows 40 kDa, and gray arrows mark NH_2_- and COOH-terminal fragments resulting from intra-molecular cleavage.

Several frame-shift mutations in human RGMc are associated with JH [4-7]. We mimicked four of these mutants in NH_2_-terminal HA-tagged mouse RGMc, and added a 6× His tag to each COOH-terminus (Fig. 2A). The mutant proteins when expressed in Cos-7 cells accumulated in culture medium, but not on the cell membrane, as they lacked a GPI attachment sequence (Fig. 2B, and data not shown). A doublet was seen in the medium of cells producing the R378X and C354X mutants, but only a single immunoreactive band with S321X and Q305X. As all protein species were detected with an antibody to the NH_2_-terminal HA tag, this result suggests that the smaller mutant proteins (and 40 kDa RGMc derived from RGMcΔGPI) may lack COOH-terminal residues (Fig. 2B). After incubation of cells with RVKR, the larger member of the protein doublet for R378X and C354X increased in abundance in the medium, and was recognized by an antibody to the COOH-terminal His tag, while the smaller of the doublet bands was not (Fig. 2C). Similar results were seen for RGMcΔGPI (Fig. 2C). In contrast, detection of S321X and Q305X with the His antibody was constant and was unaffected by RVKR. Taken together with observations in Fig. 1, the results with truncation mutants demonstrate that PC activity potentially removes a COOH-terminal segment of RGMc, and indicate that the cleavage site is located between amino acids 321 and 354 of the mouse protein, in agreement with identification of a conserved PC motif between residues 318 and 327 (Fig. 1A).

### Altered processing of newly synthesized RGMc in the presence of PC inhibitor

**Figure 3:**
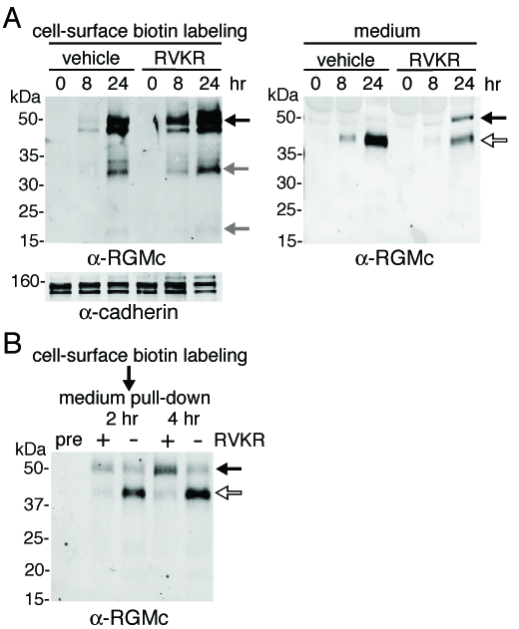
**Altered processing of newly synthesized RGMc by PC inhibition**. **A. **Time course of accumulation of RGMc on the cell surface and in conditioned medium after infection of Hep3B cells with Ad-HA-RGMc ± RVKR. Cell-surface proteins were labeled with non-permeable biotin (EZ-link), followed by incubation ± RVKR, and streptavidin pull-down, as described in 'Methods'. Immunoblot for cadherin measures sample loading. **B. **PC inhibition does not prevent acute release of RGMc from the cell surface. Membrane-associated RGMc was labeled with EZ-link, followed by incubation ± RVKR, and detection of soluble RGMc after streptavidin pull-down by immunoblotting. For **A **– **B**, arrows are described in legend to Fig. 1. Similar results were observed with Cos-7 cells.

To study effects of PC inhibition on RGMc biosynthesis and maturation, we infected Hep3B cells with Ad-tTA and Ad-HA-RGMc. Ad-tTA encodes a tetracycline-repressible transcriptional activator that stimulates the promoter regulating the gene for HA-RGMc. Under these conditions, full-length and heterodimeric RGMc were found on the cell membrane beginning at 8 h after viral infection and increased in abundance at 24 h. More membrane-associated RGMc was detected in RVKR-treated cells than in controls, although there was no change in the pattern of immunoreactive proteins. In culture medium 40 kDa RGMc accumulated starting at 8 h, and the 50 kDa species was only seen after incubation of cells with RVKR (Fig. 3A).

We used cell surface-labeling experiments to study the impact of PC inhibition on acute release of membrane-linked RGMc into the medium. In Hep3B cells expressing HA-RGMc, membrane proteins were labeled for 30 min with cell-impermeable biotin cross-linker followed by addition of RVKR or vehicle. Release of RGMc from the cell surface was monitored by immunoblotting after streptavidin pull-down of culture medium. Biotinylated 40 and 50 kDa RGMc were detected in medium at 2 and 4 h after labeling, and more 50 kDa RGMc was seen with RVKR, although the total amount of RGMc declined by ~35% (Fig. 3B). These results show that both 50 and 40 kDa soluble RGMc species derive from cell-associated 50 kDa RGMc, and demonstrate that inhibition of PC activity diminishes but does not prevent release of membrane-linked RGMc into extracellular fluid.

### Furin cleaves 50 kDa RGMc to produce a 40 kDa species

**Figure 4:**
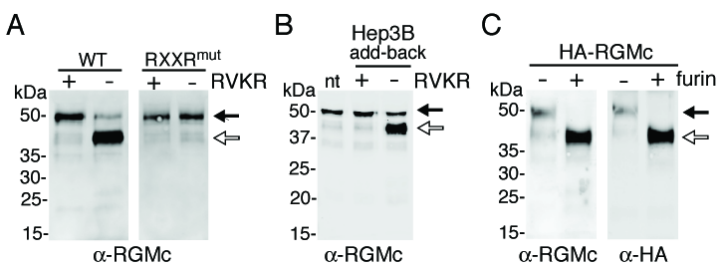
**Furin cleaves soluble RGMc to produce the 40 kDa isoform**. **A. **Only 50 kDa RGMc accumulates in culture medium of cells expressing a protein with a modified PC cleavage site. Immunoblot of soluble RGMc from conditioned medium of Cos-7 cells transfected with either wild-type (WT) RGMc or a derivative containing a mutated PC site (RXXR^mut^), after incubation ± RVKR. Similar results were observed with Hep3B cells. **B. **Hep3B cells cleave 50 kDa RGMc. RGMc was added to medium of Hep3B cells for 24 h ± RVKR, followed by immunoblotting (nt = non-treated). Identical results were seen with Cos-7 cells. **C. **Furin cleaves 50 kDa RGMc. Immunoblot shows results of *in vitro *incubation of soluble 50 kDa HA-RGMc ± recombinant furin. Antibodies are indicated. For **A **– **C**, arrows are as in legend to Fig. 1.

We destroyed putative PC recognition/cleavage sites in mouse RGMc by site-directed mutagenesis (glycine for arginine 318, alanines for arginines 321 and 324). When this mutant was expressed in Cos-7 cells, only 50 kDa RGMc was detected in conditioned medium, while wild type RGMc was processed to the 40 kDa species in the absence of RVKR, but 50 kDa RGMc was seen when the inhibitor was added (Fig. 4A).

PC activity may be found in intracellular compartments, at the membrane, and in the extra-cellular milieu [22]. To determine where cleavage of 50 kDa RGMc may occur, conditioned medium from Cos-7 cells expressing HA-RGMc was collected in the presence of RVKR, and after dialysis to remove the inhibitor, added to Hep3B cells. Following incubation for 24 h, significant conversion to 40 kDa RGMc was observed, but was not seen when RVKR was added (Fig. 4B), indicating that these cells produce PCs that act extra-cellularly.

The R-N-R-R sequence in RGMc (Fig. 1A) represents an optimal furin site [23]. In agreement with this idea, incubation of 50 kDa RGMc with recombinant furin *in vitro *led to its efficient cleavage into a 40 kDa species with an intact NH_2_-terminus, as shown by detection with both anti-HA and anti-RGMc antibodies (Fig. 4C). Thus by several criteria, 50 kDa RGMc is a PC substrate.

### Effects of iron on processing of RGMc and its release from the cell membrane

**Figure 5:**
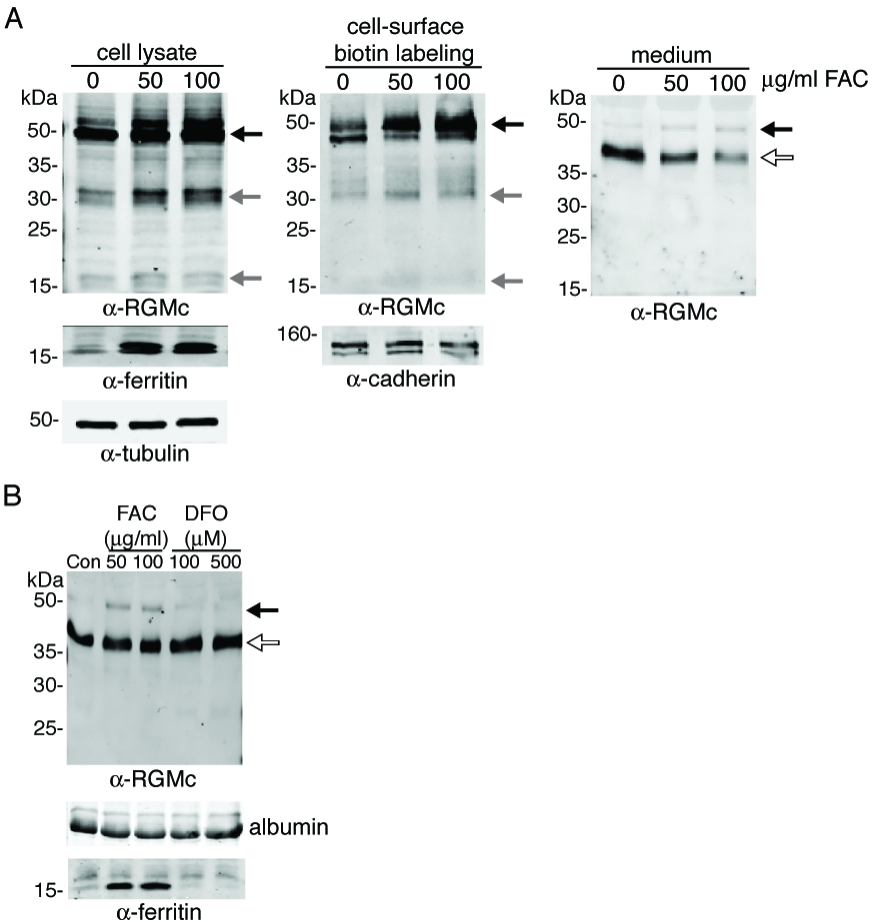
**A. Iron loading increases expression of RGMc on the cell membrane and diminishes accumulation in extra-cellular fluid**. **A. **Hep3B cells infected with Ad-HA-RGMc were incubated for 24 h with the concentrations of ferric ammonium citrate (FAC) indicated, followed by cell-surface biotin labeling, and detection of RGMc by immunoblotting in cell lysates (left panel), on the membrane after surface biotin labeling and streptavidin pull-down (middle panel), and in the medium (right panel). Similar results were observed with Cos-7 cells. **B. **Detection of RGMc by immunoblotting of conditioned medium from transiently transfected Cos-7 cells following incubation for 24 h with the concentrations of FAC or deferoxamine (DFO) indicated. Identical results were seen with Hep3B cells. For **A **– **B**, arrows are as in legend to Fig. 2.

Recent work has shown that delivery of iron to cultured cells resulted in a decline in abundance of extra-cellular RGMc 24 h later [15,24]. To address effects of iron on membrane-bound RGMc, adenoviral-infected Hep3B cells were incubated with ferric ammonium citrate, briefly labeled with cell-impermeable biotin cross-linker, and examined for accumulation of RGMc on the cell membrane and in the medium. As seen in Fig. 5A, iron loading led to a dose-dependent increase in cell surface-associated 50 kDa RGMc, and to a decline in abundance of the 40 kDa species in the medium (~50% decrease at 100 μg/ml ferric ammonium citrate). In addition, the amount of 50 kDa soluble RGMc increased. In agreement with these results, incubation of cells with the iron chelator, deferoxamine, prevented accumulation of 50 kDa RGMc in culture medium, and led to a ~30% rise in the amount of soluble 40 kDa RGMc compared with cells incubated with ferric ammonium citrate (Fig. 5B). Thus, iron delivery appears to negatively regulate release of RGMc from the membrane, and may also inhibit PC activity. In this regard, Silvestri et al have found that deferoxamine enhanced and iron loading decreased the abundance of furin in HeLa cells [25].

## Discussion

In this manuscript we show that RGMc accumulates in extra-cellular fluid of cultured cells and in mouse and human serum as 50 and 40 kDa protein species (Fig. 1). As is evident from cell-surface binding experiments, both molecules originate from cell-membrane-associated GPI-linked single-chain RGMc (Fig. 3), and the 40 kDa isoform is derived from the 50 kDa species by targeted proteolysis mediated by PCs such as furin (Fig. 4), with cleavage occurring at a site that is highly conserved among RGMc orthologues, but is absent in the paralogues, RGMa and RGMb (Fig. 1). Taken together, our results define a key role for PCs in the regulation of RGMc that has implications for the physiological effects of this critical iron-regulatory protein.

### Are there specific biological effects of different RGMc protein species?

The biological actions of RGMc are not yet fully defined. A role for RGMc in iron homeostasis is postulated based on the discovery of multiple mutations in the HJV gene in patients with JH [4-7], and on the iron overload phenotype in mice lacking *hjv *[8,9]. Loss of RGMc is associated with severe reduction in hepcidin [4,8,9], a critical negative regulator of iron absorption, placing RGMc upstream in a pathway controlling hepcidin production in the liver [3]. Cell-associated RGMc can enhance effects of BMPs to increase hepcidin gene expression, potentially through direct binding to BMP2 and 4 [18,19], but it is not known if these actions are mediated by single-chain or heterodimeric RGMc. In contrast, soluble RGMc appears to inhibit production of hepcidin mRNA [15,20]. It is not known if 50 or 40 kDa soluble RGMc proteins preferentially bind to BMPs, or if they have other actions, although a recent report showed that soluble 40 kDa RGMc blunted stimulation of hepcidin gene expression by BMP2 in cultured cells [20]. This latter observation indicates that PC activity may influence the biological actions of RGMc.

### Does iron regulate RGMc?

Serum levels of RGMc were shown to transiently increase in acutely iron-deficent rats [24], and incubation of cultured cells with holo-transferrin for 24 – 48 h caused a reduction in RGMc in the medium [15,24]. This latter effect was attributed to a decline in the extent of shedding of membrane-linked RGMc [24]. We find in agreement with these results that iron loading increased the amount of single-chain RGMc on the cell membrane, and caused a commensurate decline in accumulation of 40 kDa RGMc in the extra-cellular fluid, while the abundance of the soluble 50 kDa species increased. Additional work will be needed to define the mechanisms by which iron alters the biogenesis and processing of RGMc, although as suggested by Silvestri et al [25], one possibility may be through control of furin production.

## Conclusion

### RGMc, pro-protein convertases, and iron metabolism

**Figure 6:**
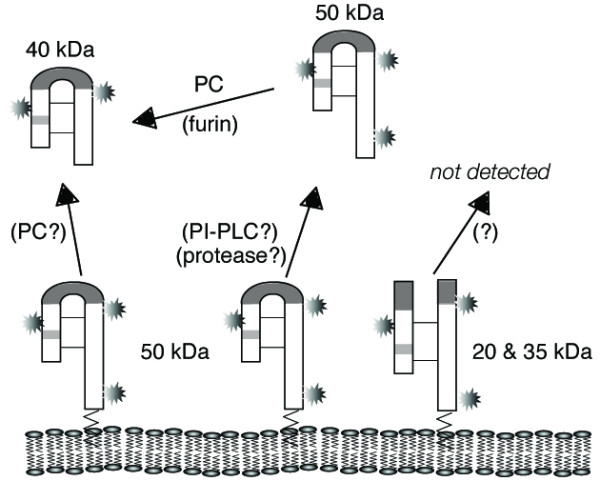
**Model for release of RGMc from the cell membrane**. Membrane-associated heterodimeric RGMc (20/35 kDa) is not found in extracellular fluid [14], and its mechanisms of processing are unknown. Full-length membrane-linked 50 kDa RGMc may be digested by a phospholipase (PI-PLC) or by an uncharacterized protease, and then by a PC to generate the 40 kDa species. Alternatively, a PC may directly cleave 40 kDa RGMc at the membrane. The starbursts represent N-linked glycosylation sites, and the thin lines, disulfide bonds.

The key observation in this manuscript is that a furin-like PC cleaves 50 kDa full-length single-chain RGMc at a conserved site to produce a 40 kDa soluble species. We find that PC activity leads to the accumulation of 40 kDa RGMc in medium of skeletal muscle cells, which endogenously synthesize RGMc [14], and in medium of several cell types expressing recombinant RGMc. Moreover, we show directly that purified recombinant furin can cleave 50 kDa RGMc *in vitro*. Recent reports from others also implicate PCs in the cleavage of RGMc [25,26]. Using Hek293 stably expressing human hemojuvelin/RGMc, Lin et al have found that a PC inhibitor blocks that appearance of a soluble form of the protein (sHJV), with a larger species (ecto-HJV), probably equivalent to 50 kDa mouse RGMc, accumulating instead [26]. Silvestri et al have made similar observations in transfected HeLa cells [25]. Our detection of both 50 and 40 kDa RGMc in human and mouse serum further supports the physiological significance of its PC-mediated proteolysis. A model summarizing our results on processing of membrane-associated RGMc is depicted in Fig. 6. As other studies indicate that furin may be involved in processing and maturation of several iron regulating proteins, including hepcidin, BMPs, and the soluble transferrin receptor [27-29], these observations when taken together imply a diverse and potentially important role for PCs in iron homeostasis.

## Authors' contributions

DK helped conceive of the study, performed experiments on RGMc purification and detection, analyzed effects of furin and iron, and helped draft the manuscript. RK-H contributed to studies on RGMc amino acid substitution and truncation mutations and helped draft the manuscript. MN generated and studied RGMc truncation mutants. PR helped conceive of the study, helped in the design and analysis of experiments, and drafted the manuscript. All authors read and approved the manuscript.
